# Cardiac patients’ perception about psychological risk factors on chest pain intensity and discomfort

**DOI:** 10.22088/cjim.9.2.204

**Published:** 2018

**Authors:** Saeid Komasi, Ali Soroush, Mozhgan Saeidi

**Affiliations:** 1 Clinical Research Development Center, Imam Reza Hospital, Kermanshah University of Medical Sciences, Kermanshah, Iran.; 2 Lifestyle Modification Research Center, Imam Reza Hospital, Kermanshah University of Medical Sciences, Kermanshah, Iran.; 3 Cardiac Rehabilitation Center, Imam Ali Hospital, Kermanshah University of Medical Sciences, Kermanshah, Iran.

**Keywords:** Cardiovascular disease, Risk factor, Perception, Pain

**Dear Editor,**

Perceived heart risk factors including psychological and non-psychological factors (components of behavioral, biological, environmental, and physiological) are as part of the mental representation of illnesses that arise from patients' health knowledge and independently can predict their health behavior (1-2). Causal beliefs and perceived risk factors are associated not only with patient's health and adjustment but also the impact on their adherence to treatment recommendations (3). Patients' cognition has a significant impact on disease course and progression during all stages of illness experience including understanding signs, looking for a reason to link the disease to it, and considering a change in an individual’s behavior (3), so it is suggested that patients with perceived psychological risk factors and who experienced more anxiety and depression be compared to other patients in the stage of secondary prevention (2, 3). Angina and suffering caused by it are other clinical representations of heart disease that almost a third of these patients complain of it even after successful revascularization and express inability to control it (4). It seems that pain intensity and discomfort, as important aspects of angina, were not affected only by cardiac event or procedure and nonphysical factors also play a role in its experience. Thus, since identifying factors associated with angina in the secondary prevention can be effective in improving the quality of life and returning patients to normal life, it seems that there is a need to conduct further studies with regard to the relationship between perceived risk factors and clinical representation of angina in heart patients.

Based on these considerations, a study was conducted to compare the chest pain severity and discomfort in heart patients with and without perceived psychological risk factors. From May to July 2015, 219 cardiac patients (23- 79 years with the mean [SD] = 58.5±9.4) after cardiac surgery were invited to participate in the study in Imam Ali Hospital of Kermanshah City (Western part of Iran). After obtaining a written informed consent to participate in the study, demographic data and medical histories of the patients were evaluated and recorded by an expert cardiologist. Then, a brief pain inventory (5), pain discomfort scale of Jensen et al. (6), and open single-item related to perceived heart risk factors (2, 4), as appropriately validated scales were provided for the patients by a clinical psychologist. Descriptive statistics and independent t-test were used to compare the differences between the two groups in terms of pain intensity and discomfort. All statistical analysis was performed using SPSS 20 software.

According to descriptive data, 63% of patients were males, 89.4% married, 34.7% housekeepers, 32.2% self-employed, 8.2% employees, and 21.9% retires. In terms of level of education, 70.3% were below high school level 18.3% high school graduate and 11.4% with a college degree. In addition, the results show that 118 and 110 people respectively are with the perceived non-psychological and psychological risk factors. The means (SD) of pain intensity respectively were 3.751.94 and 4.321.86 for the patients with perceived non-psychological and psychological risk factors. Also, the means (SD) of pain discomfort respectively were 9.486.52 and 12.307.58 for patients with the perceived non-psychological and psychological risk factors. In relation to the main analysis, the independent t-test results show that there is a significant difference between the two groups in term of pain intensity (P=0.030) and discomfort (P=0.004) and the patients with perceived psychological risk factors indicate more pain severity and discomfort compared with the patients with perceived non-psychological risk factors. Our results showed that the severity of pain and pain discomfort are more in patients with perceived psychological risk factors compared with those patients with perceived non-psychological risk factors. In this regard, the results of the two studies showed that the patients with perceived psychological risk factors experience more anxiety and depression compared to others (2, 3). Since the patient's perception of illness directly linked to actual risk factors of disease (1), and considering the relationship between chest pain and psychological symptoms, it seems that the identification of patients perceived risk factors can play an important role in screening patients suffering from angina. Therefore, there is the possibility that we can quickly recognize and control the psychological symptoms (3) and discomfort or pain through changing the patients’ perception of disease risk factors in the stage of secondary prevention.
